# Supplementary material for: Immunogenicity Analysis of the Recombinant Plasmodium falciparum Surface-Related Antigen in Mice
Source: Pathogens. 2022 May 7;11(5):550. doi: 10.3390/pathogens11050550 (PMC9145071; doi:10.3390/pathogens11050550)
Supplement: Supplementary file 1 [file pathogens-11-00550-s001.zip › pathogens-1631836-supplementary.pdf]

Supplementary Table S1 List of primer used in this study

Gene	Forward primer	Reverse primer
**F1a**	5’-GCTGATATCGGATCC AACAACAAAGACAATCATAAT AAAA-3’	5’-GTGGTGGTGCTCGAG CTTATCGTCGTCATCCTTGTAATC TGAATCGGTCTCGTTATTTGT-3’
**F2a**	5’-GCTGATATCGGATCC AGTAACAAAGAAAAGCACAAA TACT-3’	5’-GTGGTGGTGCTCGAG CTTATCGTCGTCATCCTTGTAATC TACTTGATTATTATGGTCATTGTTG-3’
**F3a**	5’-GCTGATATCGGATCC TCGAATAATAAAAAGAAGAAG AAGA-3’	5’-GTGGTGGTGCTCGAG CTTATCGTCGTCATCCTTGTAATC TGTAAGAATCACCTCGTAGCC-3’

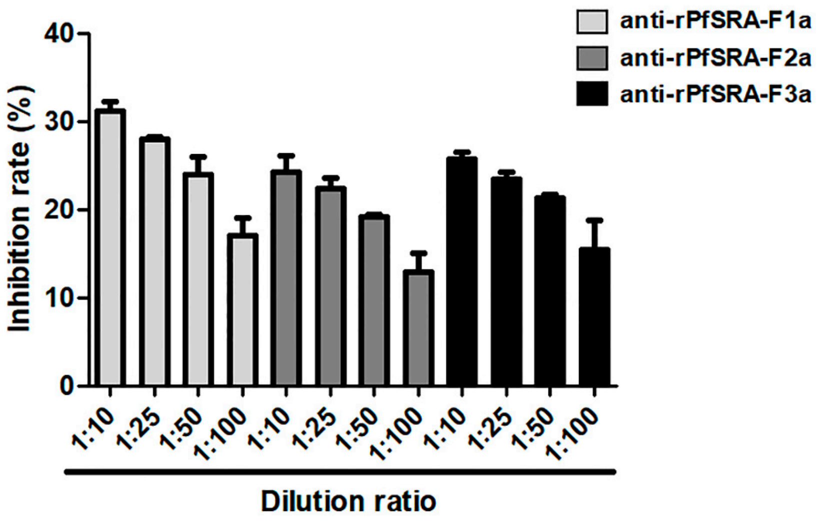

Supplementary Figure S1 Inhibition rate of anti-rPfSRA
